# Editorial: Phonology in the Bilingual and Bidialectal Lexicon

**DOI:** 10.3389/fpsyg.2017.00507

**Published:** 2017-04-04

**Authors:** Isabelle Darcy, Annie Tremblay, Miquel Simonet

**Affiliations:** ^1^Department of Second Language Studies, Indiana UniversityBloomington, IN, USA; ^2^Department of Linguistics, University of KansasLawrence, KS, USA; ^3^Department of Spanish and Portuguese, University of ArizonaTucson, AZ, USA

**Keywords:** second-language speech, bilingual and bidialectal lexicon, spoken word recognition, phonological knowledge, orthographic knowledge

One critical step when trying to comprehend a spoken message is to identify the words that the speaker intended. To recognize spoken words, listeners continuously attempt to map the incoming speech signal onto lexical representations stored in memory (McClelland and Elman, 1986; Norris, 1994): Words that partially overlap with the signal are activated until the lexical candidate that best matches the input wins over its competitors, a process known as *lexical competition*. Models of spoken-word recognition, most of which are based on native listener behavior, assume that lexical representations are stable, and contain at least the phonological form of words in citation. While lexical representations likely also contain other forms, for example the reduced forms found in conversational speech, it is a matter of debate whether native listeners encode spoken words exclusively as phonetically detailed exemplars (Johnson, 1997; Goldinger, 1998) or whether phonological abstraction also takes place (McQueen et al., 2006). Another assumption of models of native spoken-word recognition is that, under normal circumstances, listeners' perception of the input is optimal and faithful to the signal: Accurate lexical representations are easily contacted, and an optimal set of candidates is activated for quick lexical selection.

When applied to a later-learned second language (L2), two central premises of native spoken-word recognition models are compromised: (i) the premise that listeners' perception of the incoming speech signal is optimal; and (ii) the premise that listeners' lexical representations are accurate. L2 listeners are less successful at mapping the input to lexical representations, because they tend to perceive speech through their native-language (L1) phonetic categories and phonological representations. As a result, L2 listeners activate *more* and/or *different* lexical candidates than would native listeners (Broersma and Cutler, 2011). L2 listeners' knowledge of two languages further inhibits word recognition, as words from both lexicons are activated (Marian and Spivey, 2003). L2 listeners' perceptual difficulties in turn lead to the development of inaccurate or incomplete lexical representations. The fact that L2 listeners are often exposed to the orthographic form of words *before* they hear these words makes it difficult to determine the content of their lexical representations. Another relevant question is the potentially asymmetrical relationship between L2 listeners' lexical representations and their production of the same words. Thus, for L2 listeners, the links between perception, lexical representations, orthography, and production are all but clear. Even for simultaneous bilinguals, important questions remain about the specificity and interdependence of bilinguals' lexical representations and the factors influencing cross-language word activation.

This Frontiers Research Topic seeks to further our understanding of the factors that determine how bilinguals recognize and encode spoken words in the mental lexicon, with focus on the mapping between the input and lexical representations, and on the quality of lexical representations. Our call for papers resulted in 12 original contributions that represent a range of perspectives into the L2 mental lexicon and the interfaces between domains. The articles in this collection all present empirical research that revolves around three major themes.

The first theme targets interfaces and the multidirectional relationships between perception, lexical encoding, orthographic knowledge, and production. Four contributions fall under this theme. Amengual examines the interface between production and lexical encoding, and shows that even if a phonemic contrast is not part of the learner's lexical representations, it can be made in production. Cook et al. investigate the quality of phonological representations in the mental lexicon. They show that even when phonological contrasts can be perceived, learners' representations are less detailed than those of native speakers. The authors conclude that learners experience both fuzzy lexical representations and fuzzy form-to-meaning mappings. Choi et al. examine the interplay of information structure, meaning, and phonetics in the realization of word-final codas. They suggest that the L2 phonetic system can be better understood through an investigation of the phonetics–prosody interface that is further modulated by information structure and by the L2 speakers' L1 experience. Hayes-Harb and Cheng examine the interface between orthographic knowledge and the learning of new words. They show that, when establishing lexical representations, learners may need to suppress familiar orthographic information that can have interfering effects.

The second theme involves lexical access in the L1 and L2, and how it is impacted by the L1 and the developing L2 phonological systems. Three contributions fall under this theme. Freeman et al. show that L1 phonotactic knowledge impacts lexical searches during L2 word recognition. In that study, L1-Spanish L2-English bilinguals accessed their L1 Spanish phonotactic constraints during English comprehension, increasing lexical competition by activating both lexicons. A similar point is made in Broersma et al., who provide evidence for the occurrence of cross-language lexical competition in the speech of fluent Welsh-English early bilinguals: They report both facilitative and inhibitory effects in the production of cognates. The authors suggest that the shared phonological form of cognates may facilitate processing at the word-form level but result in lexical competition at the lexical-semantic level. Finally, Tremblay et al. demonstrate that L1–L2 similarities can *interfere* with segmentation processes during L2 word recognition: They show that the similarities between the prosodic systems of French and Korean make it more difficult for L1-Korean L2-French listeners to distinguish the two systems and *learn* to use the appropriate prosodic cues to word boundaries in French as compared to proficiency- and experienced-matched L1-English L2-French listeners.

The third theme deals with the speech dimensions that learners must learn to pay attention to, and how learners develop perceptual sensitivities to dimensions that matter for the purpose of lexical acquisition. Five contributions fall under this theme. Bijeljac-Babic et al. present data about bilingual infants simultaneously acquiring German and French. They show that a trochaic bias found in monolingual German infants (but not in French monolingual infants) emerges at the same time in French-German infants, and that the amount of exposure to one or the other language has little impact on the emergence of the bias. Singh et al. examine phonological variation that is lexically relevant in one language but irrelevant in the other. They show that 12-to-13-month-old bilingual infants can bind tone to meaning in Mandarin words while disregarding tone variation in English words; in contrast, monolingual Mandarin learners did not integrate tones and word meanings at the same age. Their results suggest that, early on, infants selectively adjust which speech dimensions are relevant for lexical acquisition. Blanco et al. examine the possibility that adult bilinguals have more detailed phonological representations as a result of having to keep their two languages apart and having a more variable input on which to build these representations. Barrios et al. investigate how bilingual adults learn to reorganize their perceptual sensitivity to establish sound mappings that differ across their languages. They show that bilinguals are capable of establishing new mappings to phonemes for familiar phones. Finally, Escudero et al. deal with cross-situational novel-word learning in adults, also comparing monolinguals to bilinguals, and showing that bilinguals are more accurate than monolinguals at resolving conflicting information.

All the contributions focused on bilingual rather than bidialectal listeners, but similar issues could have been raised for bidialectal listeners. We might expect similarities between bilingual and bidialectal word recognition (e.g., cross-language activation), but also differences. For instance, bidialectal listeners may experience less difficulty than bilinguals in mapping the input to lexical representations and/or more phonetic interference across the two languages due to the greater phonetic similarity of the two dialects (relative to two languages). Future research should provide a detailed examination of bidialectal word recognition, which has received very limited attention.

The contributions in this Research Topic have provided diverse and broad-ranging insights from various perspectives into bilinguals' mapping of the speech signal onto lexical representations and the quality of their lexical representations. We hope that they will inspire much needed research in this exciting area.

## Author contributions

All authors listed have made substantial, direct and intellectual contribution to the work, and approved it for publication.

### Conflict of interest statement

The authors declare that the research was conducted in the absence of any commercial or financial relationships that could be construed as a potential conflict of interest.
